# The Knowledge of Colorectal Cancer Symptoms and Risk Factors among 10,078 Screening Participants: Are High Risk Individuals More Knowledgeable?

**DOI:** 10.1371/journal.pone.0060366

**Published:** 2013-04-03

**Authors:** Martin C. S. Wong, Hoyee W. Hirai, Arthur K. C. Luk, Thomas Y. T. Lam, Jessica Y. L. Ching, Sian M. Griffiths, Francis K. L. Chan, Joseph J. Y. Sung

**Affiliations:** 1 Institute of Digestive Disease, Faculty of Medicine, Chinese University of Hong Kong, Hong Kong SAR, China; 2 School of Public Health and Primary Care, Faculty of Medicine, Chinese University of Hong Kong, Hong Kong SAR, China; Centro di Riferimento Oncologico, IRCCS National Cancer Institute, Italy

## Abstract

**Objectives:**

We tested the *a priori* hypothesis that *self-perceived* and *real presence*s of risks for colorectal cancer (CRC) are associated with better knowledge of the symptoms and risk factors for CRC, respectively.

**Methods:**

One territory-wide invitation for free CRC screening between 2008 to 2012 recruited asymptomatic screening participants aged 50–70 years in Hong Kong. They completed survey items on *self-perceived* and *real presences* of risks for CRC (advanced age, male gender, positive family history and smoking) as predictors, and knowledge of CRC symptoms and risk factors as outcome measures, respectively. Their associations were evaluated by binary logistic regression analyses.

**Results:**

From 10,078 eligible participants (average age 59 years), the mean knowledge scores for symptoms and risk factors were 3.23 and 4.06, respectively (both score range 0–9). Male gender (adjusted odds ratio [AOR] = 1.34, 95% C.I. 1.20–1.50, p<0.01), self-perception as not having any risks for CRC (AOR = 1.12, 95% C.I. 1.01–1.24, p = 0.033) or uncertainty about having risks (AOR = 1.94, 95% C.I. 1.55–2.43, p<0.001), smoking (AOR 1.38, 95% C.I. 1.11–1.72, p = 0.004), and the absence of family history (AOR 0.61 to 0.78 for those with positive family history, p<0.001) were associated with poorer knowledge scores (≤4) of CRC symptoms. These factors remained significant for knowledge of risk factors.

**Conclusions:**

Male and smokers were more likely to have poorer knowledge but family history of CRC was associated with better knowledge. Since screening of these higher risk individuals could lead to greater yield of colorectal neoplasm, educational interventions targeted to male smokers were recommended.

## Introduction

Colorectal cancer (CRC) is the third most common malignancy and the fourth leading cause of mortality worldwide, accounting for 8% of all cancer deaths [1]. Whilst it remains prevalent in Western countries, the past decade has witnessed a dramatic increase in incidence in many Asia Pacific countries, including China, Japan, Korea, Singapore and Taiwan [2]. In 2008 there were 4,335 newly diagnosed cases of CRC in Hong Kong [3], accounting for 16.7% of all new cancer cases and which have doubled over the past two decades.

Screening for CRC using fecal occult blood tests (FOBTs) has been shown to reduce the mortality of CRC by up to 33% [4]–[6]. The US Preventive Task Force and the Asia Pacific consensus statements have recommended FOBT as one of the screening tools for CRC screening [7], [8]. Notwithstanding, adherence to screening and uptake rates were still low even in developed countries [9], [10].

A recent multi-center, international study involving 14 countries or regions in the Asia Pacific region reported considerable deficiencies in knowledge of CRC symptoms and risk factors, and suggested that this could lead to poor uptake of CRC screening tests [11]. This is compatible with another interview-based survey conducted in an ethnically diverse population aged 30–70 years, which found that the overall knowledge of CRC was a significant predictor of intent to participate in CRC screening [12]. Knowledge of CRC symptoms has been identified as a powerful predictive factor of having received a CRC screening test [13]. Another population-based survey conducted among more than 1,000 residents in Hong Kong also showed that the knowledge of CRC symptoms and risk factors were low, but both were positively associated with CRC testing [14]. The importance of having good knowledge of CRC on subsequent uptake and compliance of screening has been widely recognized [15]–[17].

Nevertheless, there are presently few reports on determinants of CRC knowledge. The objectives of this study were to evaluate the factors associated with poor symptom and risk factor knowledge of CRC. Screening based on risk for CRC has been shown to be the most cost-effective approach [18] and could bring forth many potential advantages, including that higher risk individuals could be better motivated to attend screening programmes [19]. Hence, we also tested the *a priori* hypothesis that *self-perceived* and the *real presence* of risks for CRC was associated with better knowledge of CRC. Older age, male gender, family history of CRC and smoking were considered as these risks. If this hypothesis was rejected, it would imply the need for more educational initiatives to enhance knowledge of CRC for these high risk groups since it is more likely for them to accept screening invitations.

## Materials and Methods

### Ethics Statement

This study was approved by the Clinical Research Ethics Committee of the Chinese University of Hong Kong, and all the study participants gave written informed consent.

### Setting and Screening Participants

A bowel cancer screening centre was established in May 2008 and provided free CRC screening for all eligible Hong Kong citizens via one media invitation, where prospective participants could enrol via e-mails, telephone, faxlines and walk-in. The details about this setting have been described elsewhere [20], [21]. Briefly, this centre invited all self-referred screening participants aged 50–70 years who (1). were asymptomatic of CRC; (2). had not undergone any CRC screening in the past 5 years; and (3). had no contraindications for colonoscopy in the study period 2008–2012. They were given a choice of annual, fecal immunochemical test (FIT; Hemosure) for up to 5 years, or one direct colonoscopy. Before programme enrolment, they were invited to complete a self-administered questionnaire. For less literate participants, the centre staff read the question items word-by-word to facilitate survey completion.

### The Survey Instruments and Measurements

The survey items were developed by a thorough literature review and revised by a panel of epidemiologists, psychologists and clinicians. They were further piloted tested and validated, and have been used in a previous survey study conducted in various Asia Pacific countries [11], an interview-based questionnaire study in out-patient clinics in Australia [12], and also a territory-wide telephone survey in Hong Kong [14]. The questionnaire consist of items assessing the participants’ knowledge of symptoms and risk factors for CRC, respectively. The respondents were asked “What are the symptoms of bowel cancer?” and “What are the risks factors for bowel cancer?” The questions scored answers on a list undisclosed to the respondents, and each correct response scored one point based on answer keys which are universally agreed, evidence-based and guideline-accepted. The correct answers for CRC symptoms included per rectal bleeding; mucus in stool; change of bowel habit; diarrhea or constipation; abdominal or anal pain; gastrointestinal upset; anemic symptoms; weight loss and tiredness. The correct responses for risk factors for bowel cancer included advanced age; male gender; family history of CRC; low intake of fruits or vegetables; high intake of fatty food; frequent intake of meat; obesity; smoking; and certain types of bowel diseases. Both knowledge scores ranged from 0 (poorest) to 9 (best). The questionnaire also recorded demographic information, including age, sex, educational levels, marital status, occupation, monthly household income, self perceived risks of CRC, family history of CRC (no vs. first degree relatives vs. second degree relatives vs. others), perceived necessity of CRC screening for people aged 50 years or older, smoking (current smokers vs. non-smoker/ex-smokers) and body mass index (BMI). To assess self perceived risks of CRC, the survey asked “Do you perceive yourself as currently having any risk factors for CRC?” and the respondents could choose “yes”, “no” or “unsure”. The participants were also asked “How much need do you perceive people aged 50 years or older should undergo regular CRC screening?” and they were provided with the following options: “very high”; “quite high”; “quite low”; “very low”; and “unknown”. The screening participants had their body height measured by a standiometer without wearing shoes, and body weight measured on light clothing by a weighing scale which was regularly calibrated. We used the Asian definition of overweight; defined as BMI ≥23 [22].

### Outcomes and Covariates

The two outcome variables were knowledge of CRC symptoms and risk factors, respectively. The cut-off value defining poor knowledge for both variables was ≤4, dichotomized based on a recent survey defining CRC knowledge score >50% as satisfactory [23]. The variables tested for association included the self-perceived risks for CRC, positive family history of CRC involving first-degree and second-degree relatives, and current smoking. The other demographic and perceptual variables described above were covariates.

### Statistical Analysis

All categorical and continuous variables were compared according to the knowledge of CRC symptoms and risk factors by chi-square tests of heterogeneity and Student’s t-tests of independence, respectively. Two separate binary logistic regression models were constructed with knowledge of CRC symptoms and risk factors as outcome variables, respectively. All potential predictors and covariates were unconditionally entered into the regression analyses, and tested for interactions and collinearity. A two-sided p value of <0.05 was regarded as statistically significant.

### Sensitivity Analyses

Owing to the arbitrary nature of the cut-off value for good vs. poor knowledge, we separately defined scores of ≤3 and ≤5 for both knowledge measures as poor. The regression analyses were re-conducted to detect any differences in the significance of the associated factors.

## Results

### Participant Characteristics

A total of 10,078 consecutive participants completed the surveys. Their average age was 59 years with a female proportion of 56.4% (**Table 1**). In all, 66% of the respondents were aged 50 to 59 years. Most attained secondary educational level (56.9%) and were married/cohabited (84.5%). One third of the participants had full time jobs and 57.4% of them had monthly household income lower than HK$20,000 (US$2,571). 38.2% of the respondents perceived themselves as at risk for CRC. The majority of them did not have family history of CRC (57.7%), and most perceived CRC screening for people aged ≥50 years as “very” or “quite” necessary (83.4%). Only 5.1% were current smokers, and 50.7% were overweight (BMI ≥23).

**Table 1:** Participant Characteristics (N = 10,078).

	No. of Participants^a^	Percentage^b^
***Age (years)***		
50–54	3408	33.8
55–59	3244	32.2
60–64	2280	22.6
65–70	1136	11.3
***Gender***		
Male	4384	43.5
Female	5689	56.4
***Educational level***		
Primary or below	2747	27.3
Secondary	5739	56.9
Tertiary or above	1576	15.6
***Marital status***		
Married/cohabit	8514	84.5
Single/divorced/widowed/others	1546	15.3
***Occupational status***		
Full time	3609	35.8
Part time or retired	3424	34.0
Housewife and others	3030	30.1
***Monthly household income ($US)***		
<1285$	2932	29.1
1285$–2571$	2856	28.3
2571$–3856$	1428	14.2
3856$–5141$	665	6.6
>5142$	611	6.1
Refused to answer	1572	15.6
***Self perceived risk of CRC*** *		
At risk	6873	38.2
Not at risk	2552	25.3
Not sure	608	6.0
***Family history of CRC*** *		
Nil	5714	57.7
First degree relatives	1313	13.0
Second degree relatives	1242	12.3
Others	1709	17.0
***Perceived Necessity of CRC*** * ***screening for people aged ≥50***		
Very or quite necessary	8402	83.4
Not very necessary or unnecessary	344	3.4
Not sure	1315	13.0
**Smoking**		
Nil/ex-smoker	9541	94.7
Current smoker	512	5.1
**Body Mass Index (kg/m^2^)**		
<23	4358	43.2
≥23	5107	50.7

* CRC: Colorectal Cancer.

### Knowledge Scores on Symptoms and Risk Factors for CRC

The mean knowledge scores for symptoms and risk factors were 3.23 and 4.06, respectively. From bivariate analyses, higher symptom scores were reported among the younger subjects, female, those with higher monthly household income, people who perceived themselves as having risks for CRC, participants with family history of CRC among their first and second degree relatives, respondents who perceived CRC screening as necessary among people aged ≥50 years, non-smokers/ex-smokers, and those with BMI <23 (all p<0.001) (**Table 2**). These observations were similar for knowledge scores of CRC risk factors, except participants’ gender (p = 0.231) and BMI (p = 0.940) which did not attain statistical significance.

**Table 2:** Knowledge scores on symptoms and risk factors of Colorectal Cancer (CRC).

	Symptoms			Risk factors		
	No.	Mean Score (S.D.)	p	No.	Mean Score (S.D.)	p
***Age (years)***						
50–54	3407	3.90 (2.08)	<0.001	3407	4.16 (2.07)	<0.001
55–59	3244	3.75 (2.16)		3244	4.09 (2.18)	
60–64	2280	3.49 (2.27)		2279	3.90 (2.33)	
65–70	1136	3.39 (2.38)		1136	3.97 (2.53)	
***Gender***						
Male	4384	3.55 (2.17)	<0.001	4382	4.03 (2.27)	0.231
Female	5688	3.82 (2.20)		5689	4.08 (2.20)	
***Educational level***						
Primary or below	2747	3.02 (2.20)	<0.001	2747	3.40 (2.33)	<0.001
Secondary	5738	3.89 (2.12)		5737	4.20 (2.12)	
Tertiary or above	1576	4.23 (2.16)		1576	4.71 (2.11)	
***Marital status***						
Married/cohabit	8514	3.70 (2.19)	0.753	8512	4.06 (2.22)	0.597
Single/divorced/widowed/others	1545	3.72 (2.20)		1546	4.09 (2.25)	
***Occupational status***						
Full time	3608	3.71 (2.09)	0.719	3608	4.03 (2.15)	0.204
Part time or retired	3424	3.68 (2.25)		3423	4.11 (2.31)	
Housewife and others	3030	3.72 (2.24)		3030	4.04 (2.22)	
***Monthly household income ($US)***						
<1285$	2932	3.54 (2.28)	<0.001	2931	3.93 (2.33)	<0.001
1285$–2571$	2855	3.71 (2.09)		2856	4.04 (2.19)	
2571$–3856$	1428	3.82 (2.09)		1428	4.16 (2.07)	
3856$–5141$	665	3.90 (2.06)		664	4.19 (2.05)	
>5142$	611	4.27 (2.16)		611	4.53 (2.07)	
Refused to answer	1572	3.59 (2.32)		1572	4.02 (2.33)	
***Self perceived risk of CRC*** *						
At risk	6872	3.86 (2.14)	<0.001	6872	4.34 (2.14)	<0.001
Not at risk	2552	3.66 (2.13)		2552	3.80 (2.11)	
Not sure	608	2.05 (2.31)		608	2.02 (2.46)	
***Family history of CRC*** *						
Nil	5813	3.41 (2.20)	<0.001	5812	3.86 (2.28)	<0.001
First degree relatives	1313	4.20 (2.11)		1313	4.33 (2.09)	
Second degree relatives	1242	4.22 (2.16)		1242	4.37 (2.16)	
Others	1709	3.96 (2.09)		1709	4.31 (2.12)	
***Necessity of CRC*** * ***screening for*** ***people aged ≥50***						
Very or quite necessary	8401	3.89 (2.14)	<0.001	8400	4.27 (2.16)	<0.001
Not very necessary orunnecessary	344	3.22 (2.25)		344	3.45 (2.32)	
Not sure	1315	2.61 (2.16)		1315	2.87 (2.20)	
**Smoking**						
Nil/ex-smoker	9540	3.74 (2.18)	<0.001	9539	4.08 (2.22)	<0.001
Current smoker	512	2.97 (2.25)		512	3.67 (2.36)	
**Body Mass Index (kg/m^2^)**						
<23	4358	3.89 (2.14)	<0.001	4358	4.07 (2.13)	0.940
≥23	5106	3.59 (2.20)		5105	4.07 (2.27)	

* CRC: Colorectal Cancer.

### Factors Associated with Poor Knowledge Scores

From multivariate regression analyses, factors found to be significantly associated with **poorer knowledge** **of CRC symptoms** included **male gender** (adjusted odds ratio [AOR] = 1.343, 95% C.I. 1.203–1.498, p<0.001); **primary educational level** (AOR = 0.489 to 0.599 for secondary and tertiary levels, both p<0.001); **full time job status** (AOR for other job status = 0.858 to 0.869, both p<0.05); **self-perception as not being at risk for CRC** (AOR = 1.118, 95% C.I. 1.009–1.238, p = 0.003); **uncertainty about CRC risks** (AOR = 1.942, 95% C.I. 1.550–2.433, p<0.001); **absence of family history** (AOR for any relatives having past medical history of CRC = 0.614 to 0.625, both p<0.001); **perception of CRC screening for people aged ≥50 years as “not very necessary” or “unnecessary”** (AOR = 1.341, 95% C.I. 1.036–1.735, p = 0.026) or **being “unsure”** (AOR = 2.083, 95% C.I. 1.788–2.427, p<0.001); **current smokers** (AOR = 1.381, 95% C.I. 1.106–1.723, p = 0.004); and those who were **overweight or obese** (AOR = 1.140, 95% C.I. 1.043–1.245, p = 0.004). These factors remained significant when knowledge of CRC risk factors was the outcome variable, except for smoking which did not reach statistical significance and that participants with BMI <23 were associated with poorer knowledge (**Table 3**).

**Table 3:** Factors associated with poorer knowledge scores on symptoms and risk factors of Colorectal Cancer (CRC).

	Symptoms		Risk factors	
	Adjusted odds ratio (95% C.I.)	p	Adjusted odds ratio (95% C.I.)	p
***Age (years)***				
50–54	1.00 (referent)		1.00 (referent)	
55–59	1.035 (0.930–1.152)	0.530	0.968 (0.872–1.076)	0.549
60–64	1.091 (0.960–1.241)	0.182	1.018 (0.898–1.154)	0.779
65–70	1.074 (0.903–1.276)	0.421	0.865 (0.731–1.023)	0.089
***Gender***				
Female	1.00 (referent)		1.00 (referent)	
Male	1.343 (1.203–1.498)	<0.001	1.195 (1.073–1.331)	0.001
***Educational level***				
Primary or below	1.00 (referent)		1.00 (referent)	
Secondary	0.599 (0.534–0.670)	<0.001	0.598 (0.536–0.667)	<0.001
Tertiary or above	0.489 (0.418–0.571)	<0.001	0.404 (0.347–0.471)	<0.001
***Marital status***				
Married/cohabit	1.00 (referent)		1.00 (referent)	
Single/divorced/widowed/others	1.009 (0.888–1.145)	0.895	0.972 (0.858–1.101)	0.654
***Occupational status***				
Full time	1.00 (referent)		1.00 (referent)	
Part time or retired	0.869 (0.772–0.977)	0.019	0.837 (0.746–0.939)	0.002
Housewife and others	0.858 (0.753–0.979)	0.023	0.832 (0.731–0.947)	0.005
***Monthly household income ($US)***				
<1285$	1.00 (referent)		1.00 (referent)	
1285$–2571$	0.965 (0.855–1.088)	0.559	1.032 (0.917–1.160)	0.604
2571$–3856$	0.996 (0.859–1.154)	0.9523	0.975 (0.844–1.125)	0.729
3856$–5141$	0.936 (0.772–1.135)	0.502	1.014 (0.840–1.224)	0.887
>5142$	0.805 (0.657–0.988)	0.038	0.930 (0.760–1.138)	0.481
Refused to answer	0.938 (0.814–1.081)	0.376	0.915 (0.796–1.051)	0.211
***Self perceived risk of CRC***				
At risk	1.00 (referent)		1.00 (referent)	
Not at risk	1.118 (1.009–1.238)	0.033	1.545 (1.396–1.709)	<0.001
Not sure	1.942 (1.550–2.433)	<0.001	2.466 (1.986–3.063)	<0.001
***Family history of CRC***				
Nil	1.00 (referent)		1.00 (referent)	
First degree relatives	0.625 (0.549–0.711)	<0.001	0.943 (0.829–1.073)	0.372
Second degree relatives	0.614 (0.538–0.702)	<0.001	0.806 (0.706–0.920)	0.001
Others	0.781 (0.693–0.880)	<0.001	0.800 (0.712–0.899)	<0.001
***Necessity of CRC screening for people aged ≥50***				
Very or quite necessary	1.00 (referent)		1.00 (referent)	
Not very necessary or unnecessary	1.341 (1.036–1.735)	0.026	1.731 (1.340–2.236)	<0.001
Not sure	2.083 (1.788–2.427)	<0.001	2.673 (2.301–3.105)	<0.001
**Smoking**				
Nil/ex-smoker	1.00 (referent)		1.00 (referent)	
Current smoker	1.381 (1.106–1.723)	0.004	1.037 (0.846–1.269)	0.729
**Body Mass Index (kg/m^2^)**				
<23	1.00 (referent)		1.00 (referent)	
≥23	1.140 (1.043–1.245)	0.004	0.885 (0.812–0.965)	0.006

* CRC: Colorectal Cancer.

There existed no interactions among the potentially independent variables, and there were no multicollinearity detected in the two regression models. In addition, sensitivity analyses using scores ≤3 and ≤5, respectively, to dichotomize good vs. poor knowledge did not detect any heterogeneity of any associated factors reported above.

## Discussion

From this survey involving more than 10,000 self-referred screening participants, their knowledge levels on CRC symptoms and risk factors were found to be relatively low. Male subjects, people who did not perceive themselves as having risks for CRC, and participants who had no family history of CRC had poorer knowledge of CRC symptoms and risk factors. Smokers had poorer knowledge scores on CRC risk factors. Age was not a significant correlate, and the association between BMI and knowledge was equivocal. Other significant factors identified included educational level, occupational status, perception of necessity of CRC screening for people aged ≥50 years and BMI.

Worldwide, a substantial body of evidence pointed towards a low level of knowledge of CRC in many countries. The Asia Pacific Working Group in Colorectal Cancer conducted a multinational survey in various Asia Pacific regions and found that the median symptom and risk factor knowledge scores ranged from 0–4 out of 9, with quite a number of regions scoring 0 [11]. Another survey among moderate risk patients in West Malaysia reported that only 4.1% had good knowledge of CRC and its screening [24]. Other studies on CRC knowledge among indigenous Western Australians [15], Iranians [25], American Indians [26], an ethnically diverse population in Australia [16] and Hong Kong [23] also found low levels of knowledge of CRC, including awareness of its symptoms and risk factors. Few studies have, however, identified the independent predictors of poor knowledge, and the sample sizes in these studies are not large. The present study is an evaluation in a larger scale which assessed levels of CRC knowledge, and tested a full set of potential predictors for associations with poor knowledge.

These findings bear significant implications to clinical practice and policy-making in CRC screening programmes. We evaluated the association between knowledge levels and one’s risk for CRC, both self-perceived and self-reported. In populations where screening adherence is hindered by the lack of community awareness, inadequate healthcare advocacy and low programmatic compliance, focused educational efforts should be targeted towards people at higher risks for CRC since they are generally more motivated to undergo screening. This could lead to more cost-effective promotion of screening uptake by optimizing efficient resource utilization, particularly in relatively underprivileged countries. From the recent Asia Pacific Colorectal Screening (APCS) scoring system which stratified risk in the target population, age, gender, family history and smoking have emerged as risk factors which could successfully predict the risk of colorectal advanced neoplasia in asymptomatic Asian subjects [19]. Among these four risk factors, male gender and smoking were found to have poorer symptom and risk factor knowledge in this study. According to this scoring system, subjects aged 50 years or older with these two concomitant factors scored 4 out of 7, representing individuals having fourfold higher risk compared with the average risk group [19]. Their poorer knowledge is anticipated to further minimize their likelihood of screening, and should be the target group with a top priority for health education.

A large proportion of respondents (42.3%) had family history of CRC. This finding is expected as this screening programme was self-referred and people with family members having CRC might be more attracted to attend to screening initiatives. Positive family history of CRC among first and second degree relatives was associated with higher knowledge levels. According to the Health Belief Model (HBM), motivation to undertake health behavior is influenced by an individual’s perceived susceptibility, perceived severity, perceived benefit, perceived barriers and cues to action. Subjects with family members having CRC could have all these facilitators of screening, but might experience psychological barriers like apprehension to face the higher-than-average possibility of positive screening results [14]. Those with family history in a first-degree relative have more than twofold higher risks for colorectal neoplasia [19], and their participation in screening would lead to higher yield. Indeed, a further analysis of this study showed that people with positive family history had higher self-perceived risks for CRC. The proportions of subjects who perceived themselves at risks for CRC were 79.2% (first degree relatives having CRC), 69.1% (second degree relatives), 69.1% (other family members) and 65.8% (absence of any family history of CRC) (p<0.001). Therefore, as they are already equipped with good knowledge of CRC and motivators for screening, intervention in the form of educational initiatives should not be the primary strategy to promote screening. Several studies consistently reported denial of CRC risk among individuals with positive family history, leading to delay or failure to seek CRC screening [16]. It has been suggested that community members like ex-bowel cancer patients could act as lay health advisors and establish a network of peer educators, so that counseling programmes could be conducted to relieve the psychological concerns among those with positive family history. Sharing groups and information exchange sessions organized by lay health advisors from existing community networks have been proven effective to provide satisfactory emotional and instrumental support [27].

People who were employed full-time were found to have lower poorer knowledge. It might be that those with full-time employment were more occupied with their own job duties, and could be less aware of educational initiatives on CRC, and hence less knowledgeable. Also those with self-perceived risk of CRC were found to be more knowledgeable on CRC. High subjective perception of risk has previously been demonstrated as an independent predictor for CRC screening [28]. As this study showed that those who did not perceive themselves at risk or were unsure about risks had lower knowledge scores, community programmes on CRC prevention including health seminars and exhibitions should include educational sessions providing CRC risk estimation for attendees. These allow people who were initially uncertain of their risk to be informed of their estimated risks, and hence increase the likelihood for them to participate in future CRC screening programmes.

This study included a large sample size and used validated instruments for both outcome variables. We have also evaluated the association between each individual’s risk factors for CRC, instead of the calculated risks, with their knowledge levels. This approach is easier for physicians to identify patients at higher risk for poor knowledge using any of the risk factors instead of patients having higher scores. It will allow better identification of subjects who should be targeted for educational intervention. However, there are several limitations that should be addressed. First, the sampling frame included self-referred screening participants who were arguably more health-conscious and motivated for screening than the general public. Before attendance to the screening programme they could have acquired more knowledge of CRC from the media and other resources, thus their knowledge scores might not be generalizable to the general community. In addition, although the screening invitation was open to all Hong Kong residents, the screening centre is situated in only one district of Hong Kong and therefore residents living closer to the centre were more likely to attend due to geographical convenience. Also, the cross-sectional nature of this study could not establish cause-and-effect relationship because of the possibility of reverse causality. For instance, those who perceived themselves as having higher risks for CRC could actively acquire more knowledge of CRC, while higher knowledge levels could enhance one’s self-perception of CRC risks. Lastly, there were other potential confounders which have not been controlled, like prior exposure to educational activities concerning control of CRC in clinical and community settings.

Compliance with CRC screening programmes remained low and a cost-effective approach to maximize screening efficacy is to involve more high risk subjects [18]. We showed in this study that male smokers as a high risk group were less knowledgeable on CRC symptoms and risk factors, and they should be targets for more educational interventions as were people with other independent factors associated with poor knowledge. On the contrary, subjects with positive family history had satisfactory knowledge of CRC and were cognizant about their risks for CRC. The intervention strategy should be more focused on addressing their perceived psychological barriers instead of health education alone. Future research should evaluate what educational interventions are most effective and feasible for people at higher risks for poor knowledge of CRC. In addition, similar surveys should be conducted in the future in different population groups to compare findings.
